# The role of large immune complexes in anti-drug antibody development: a case study of anti-SARS-CoV-2 antibody therapeutics and co-administered mRNA vaccine

**DOI:** 10.3389/fimmu.2026.1769163

**Published:** 2026-03-10

**Authors:** Susan C. Irvin, Zhiqiang Wang, Samit Ganguly, Deepanshu Choudhary, Minita Kanagaraj, Nina Liu, Flonza Isa, Zhongqing Will He, Hong Yan, Veronica Mas Casullo, Kenneth C. Turner, John D. Davis, Michael P. Rosconi, Albert Torri, Michael A. Partridge

**Affiliations:** Regeneron Pharmaceuticals, Inc., Tarrytown, NY, United States

**Keywords:** anti-drug antibodies, COVID-19, immune complex, immunogenicity, monoclonal antibodies

## Abstract

The administration of therapeutic proteins may induce an anti-drug antibody (ADA) response which may impact pharmacokinetics, safety or efficacy. Numerous factors contribute to ADA development, such as patient population, drug sequence, formulation impurities, as well as drug dose and frequency. Here we report data from a natural experiment where ADA incidence for monoclonal antibodies (mAbs) casirivimab (CAS) and imdevimab (IMD), targeting the SARS-CoV-2 spike protein, was more than 3-fold higher in COVID-19 vaccinated participants compared to unvaccinated. Although ADA incidence to the mAbs was elevated in vaccinated participants, there was no increase in the strength or magnitude of the ADA response, despite these participants developing robust immunogenicity directed against the COVID-19 vaccine. *In vitro* studies using sedimentation velocity analytical ultracentrifugation demonstrated large complexes (ranging from 1.6 to 4 MDa) being formed between CAS+IMD and recombinant spike trimer. In addition, the substantially increased immunogenicity to CAS+IMD was only observed in participants receiving mRNA-LNP-based products, likely due to higher expression of spike protein compared to adenovirus-based products. No increase in ADA was observed in COVID-19 vaccinated participants receiving mAbs to unrelated targets, suggesting COVID-19 vaccination was not a general adjuvant. Taken together, these data suggest in participants vaccinated with mRNA-LNP-based products, the formation of large mAb-target immune complexes likely results in greater surveillance by immune cells and increased ADA development to mAbs against the same target.

## Introduction

When a therapeutic protein is administered, the host’s immune system may respond to the introduced protein. In most cases the immune response to the therapeutic protein is not clinically impactful, however, in rare cases serious safety events may occur as observed in historical case studies with recombinant human erythropoietin (epoetin) and the humanized monoclonal antibody natalizumab (1). Consequently, in clinical trials the immune responses of participants to therapeutic proteins are monitored following guidance documents from regulatory bodies (2, 3); one type of patient monitoring is performed by assessing if an antibody response against the drug develops after drug administration. The incidence of observed anti-drug antibodies (ADA) and the type of ADA (i.e., binding or neutralizing antibodies [NAb]) from clinical trial participants is then used to inform clinicians and patients via the therapeutic’s prescribing information (4).

A large case study was recently published on the immunogenicity of 2 monoclonal antibodies (mAbs), casirivimab (CAS) and imdevimab (IMD), directed against the receptor binding domain of the SARS-CoV-2 spike protein (5). These 2 non-competing, IgG1 mAbs were used as a therapeutic cocktail (CAS+IMD) in multiple different settings, including treatment of disease or prophylaxis. Due to the large size of this dataset (7,000 participants across 4 clinical trials), we were able to investigate the impact of a range of factors on the development of ADA to these mAbs, such as sample collection timing, frequency of drug administration, dose of drug, and route of drug administration.

As reported previously, there was low incidence (<11%) of ADA to CAS and IMD that developed in both disease treatment and prophylaxis settings (5). When immune responses were observed, it was usually against both mAbs (5). The concentrations of mAb in serum were similar between participants who did and did not develop immune responses to CAS and IMD, indicating no impact of ADA on pharmacokinetics (5). Furthermore, no differences in safety or efficacy to CAS+IMD were observed between these participants (5). Interestingly, the ADA incidence (but not titer) increased over time after the final drug administration (5), potentially due to low affinity B-cell responses that developed but were not detected (as ADA) until late timepoints due to affinity maturation (6, 7), and/or decreased exposure to CAS+IMD over time, leading to decreased tolerance of the foreign antigen (observed as increased ADA) (8). Frequency of administration, dose, and route of administration (i.e., IV and SC) of the mAb cocktail did not impact the incidence of immunogenicity to CAS and IMD (5).

There are numerous elements that can affect the likelihood of developing ADA and its potential clinical impact, including patient-, product-, and clinical trial-related factors (2, 3, 9, 10). Examples of risk factors include the existence of non-human sequences, low and/or intermittent dosing, and the presence of aggregates and other impurities in the drug product. Case studies have been helpful in elucidating some of the underlying causes for ADA development, with important examples highlighting the impact of aggregates and higher molecular weight complexes (11–13).

Increases in immunogenicity associated with immune complex (IC) formation have been reported extensively, including for the TNF-inhibitor mAbs, adalimumab and infliximab, which also have high ADA incidence (14–16). Separately, mRNA vaccines are known to generate substantially higher antigen concentrations than other vaccine types (14–18). Therefore, it is possible that the presence of elevated levels of viral spike protein in individuals administered mRNA-LNP-based COVID-19 vaccines also resulted in the formation of large drug:target ICs leading to enhanced uptake by antigen presenting cells (e.g., dendritic cells and macrophages). This paper demonstrates that very large mAb:target ICs (~1600 to 4000 kDa) form between the spike trimer and CAS+IMD *in vitro*, but only when both mAbs are present. This is consistent with the hypothesis that ADA development may increase when large ICs are present due to increased immune surveillance.

All of the previously reported CAS+IMD immunogenicity findings were derived from unvaccinated study participants, and therefore the exogenous target (i.e., spike protein) was not present or was present at very low levels in circulation (5). Here, we present data on the immunogenicity to CAS and IMD from study participants vaccinated with SARS-CoV-2 vaccines. Interestingly, participants that received mRNA-based vaccines had substantially higher rates of ADA than unvaccinated participants, although ADA titers and NAb rates remained low in both populations.

## Methods

### Clinical studies

As identified in **Table 1** and described by Isa, et al. (19)(**Supplementary Material**), Study COV-2118 (NCT04852978) randomized healthy participants to receive a single IV or SC dose of CAS+IMD ranging from 0.012 to 1.2 g or placebo and all participants were vaccinated with Moderna mRNA-1273 (19). The dose and route of administration of the mAb cocktail, and the timing of mAb and vaccine administration varied by participant group. The immunogenicity response to CAS and IMD in serum was monitored over time via sample collections at baseline, study day 29 (D29), and at a later cohort-specific timepoint of D169 or D183. The immunogenicity data analysis included 233 study participants.

**Table 1:** Description of Study COV-2118 and COV-2069.

Study	COV-2118 vaccine interaction	COV-2069 household contacts
Participants (N)	Healthy	Healthy
ADA Analysis Population (N)	CAS+IMD Dosed (233)	Cohort A (2,816)
Baseline SARS-CoV-2 Status	Negative	Negative
Reference	Isa, F., et al. (19)	O’Brien, M.P, et al. (20 and 21)
CAS+IMD mAb cocktail		
Dose (g)	IV: 0.012, 0.048, 0.15, 0.3, 1.2 SC: 0.06, 1.2	SC: 1.2 or placebo
mAb Administration	Single dose	Single dose
ADA Sample Collection (day)	1 (pre-dose), 29, 169 or 183	1 (pre-dose), 29, 113, 225
Vaccine		
Modality (Manufacturer)	LNP-mRNA (Moderna)	LNP-mRNA (Moderna or Pfizer-BioNTech) or Adenovirus (Janssen)
Vaccine Administration*	D1 or D15	Various

*The indicated vaccine administration day is the day when the initial vaccine dose was administered.

Study COV-2069 (NCT04452318) randomized healthy participants to receive a single SC dose (1.2 g) CAS+IMD or placebo (20, 21). For monitoring of immunogenicity against CAS and IMD, blood samples were collected from all participants at baseline and study day 29 (D29), D113, and D225. The use of approved or Emergency Use Authorized (EUA) COVID-19 vaccines was permitted after D29 when participants entered the study follow up period. Participants were enrolled in one of 2 cohorts depending on their SARS-CoV-2 infection status at screening. There was an insufficient quantity of participants who were infected at enrollment and subsequently permitted to be vaccinated, thus the *post-hoc* analysis described here is for 2,816 participants who were uninfected at enrollment and later permitted to be vaccinated (i.e., Cohort A).

### Immunogenicity Assays

In the CAS+IMD drug development program, immunogenicity responses were monitored with multiple non-quantitative assays: 3-tiered binding ADA methods specific for CAS and for IMD; and for ADA-positive samples NAb methods specific for CAS and for IMD (5). The 4 electrochemiluminescent bridging immunoassays methods were validated using a predefined set of validation parameters consistent with literature and health authority guidances including assay precision, sensitivity, drug tolerance, hook effect, specificity, recovery, robustness, and analyte stability. Anti-idiotypic antibodies to each mAb drug were used as positive controls in each method. Immunogenicity responses to each mAb were assessed in samples treated with acid to dissociated antibody: drug complexes to improve detection of ADA and NAb in the presence of drug. For the ADA assay, labeled drug (i.e., CAS or IMD) was used to create a bridge when ADA was present in a serum sample; the bound ADA was detected by an electrochemiluminescent signal from the ruthenium-labeled drug when voltage was applied to the plate. The measured electrochemiluminescence was proportional to the amount of ADA or positive control in the sample. Samples that were initially positive for ADA were then tested in the confirmatory assay to determine if the ADA response was specific to the drug. Samples that confirmed positive were then tested in the titer assay to determine the magnitude of the response. The presence of NAb in confirmed positive samples was assessed using a competitive ligand binding assay.

### ADA data analysis

The ADA analysis sets, ADA response classifications, and NAb analysis sets are similar to those previously described for the CAS+IMD studies with low ADA incidence (5).

Briefly, all participants were included in the ADA analysis sets if they received any CAS+IMD (Study COV-2118 and COV-2069) or placebo (Study COV-2069) and had at least one post-dose non-missing result. Results presented here are the ADA responses that developed after dosing of CAS+IMD.

Similarly, all participants were included in the NAb analysis sets if they received any CAS+IMD (Study COV-2118 and COV-2069) or placebo (Study COV-2069) and had at least one non-missing ADA result, and either tested negative at all ADA sampling times or tested positive with at least one non-missing NAb result after administration of CAS+IMD.

The immunogenicity responses are presented as cumulative incidence over time up to and including the indicated study visit; the data do not indicate prevalence at the study visit.

### *In vitro* analysis of complexes

To generate drug:target ICs *in vitro*, recombinant SARS-CoV-2 spike trimer (catalog number SPN-C52H3, ACROBiosystems, Newark, DE) was mixed in 1xDPBS+10%Trehalose buffer with CAS or with IMD individually at molar ratios of 1:3 (0.5 µM:1.5 µM spike trimer:mAb), and was also mixed with both mAbs (CAS+IMD) simultaneously 1:3:3 (0.5 µM:1.5 µM:1.5 µM spike trimer:CAS: IMD). Experimental controls of spike trimer alone (0.5 µM) and mAb alone (0.5 µM) were also included. Sedimentation velocity analytical ultracentrifugation (SV-AUC) was performed using an Optima AUC instrument (Beckman Coulter, Inc., Indianapolis, IN, USA) to evaluate the relative size distribution of *in vitro* complexes.

Briefly, the samples (i.e., only spike trimer or each mAb – or mixtures of spike trimer with mAb(s)) were loaded into cell assemblies with 12-mm epon double-sector centerpieces and sapphire windows. The cell assemblies were loaded in an 8-hole rotor and were incubated for 2 hours at 20 °C. To effectively monitor the particles with a large size range, a gravitational sweep method was used (22). In this method, the samples were sequentially subjected to rotor speeds of 10,000 rpm, 20,000 rpm, 28,000 rpm, 35,000 rpm and 40,000 rpm. Each speed was maintained for approximately 45 minutes, except for the final speed which was run for approximately 8 hours; absorbance data were collected at 230 nm at each speed step. Data analysis was carried out using the program, SEDFIT (version #16, National Institutes of Health) that performs size distribution analyses based on Lamm equation modeling of the sedimentation data (23, 24). SV-AUC data was loaded into SEDFIT and the scans were sorted and corrected for the speed dependent rotor stretching (22). Data were analyzed using a continuous sedimentation coefficient distribution c(s) model and c(s) distributions were plotted using GUSSI (version 1.4.2, biophysics.swmed.edu/MBR/software.html). The c(s) distributions were also used to determine the theoretical molar mass for the predominantly observed species.

The sequence of the recombinant spike trimer used to generate ICs includes the 2 proline (2P) sequence in the protein ectodomain previously identified as necessary for retaining the spike trimer’s prefusion confirmation (25). The same 2P repeat was present in the COVID-19 vaccine sequence from Moderna (i.e., version OK120841.1) and Pfizer-BioNTech (26, 27).

## Results

### Increased immunogenicity in the presence of exogenous target

A clinical study (COV-2118) was designed to determine if mAbs (CAS+IMD) directed against the SARS-CoV-2 spike protein would interfere with the humoral immune response to the viral protein after COVID-19 vaccination (28). In study COV-2118, healthy participants were randomized to receive various IV or SC doses of CAS+IMD at different times relative to mRNA vaccination, or vaccine alone. A secondary objective of the study was to assess the immunogenicity of CAS+IMD over time (19).

During the height of the pandemic and prior to the wide availability of COVID-19 vaccines, multiple other CAS+IMD clinical trials were underway in which participants were not exposed to high levels of vaccine-induced soluble target in circulation near to the time of CAS+IMD administration. Study COV-2118 therefore provided an unusual opportunity to perform an analysis of the development of CAS+IMD immunogenicity in participants when soluble target was intentionally present in blood at the time of CAS+IMD administration and could be compared to the prior CAS+IMD studies in which soluble target was absent or at low concentrations systemically.

**Figure 1:**
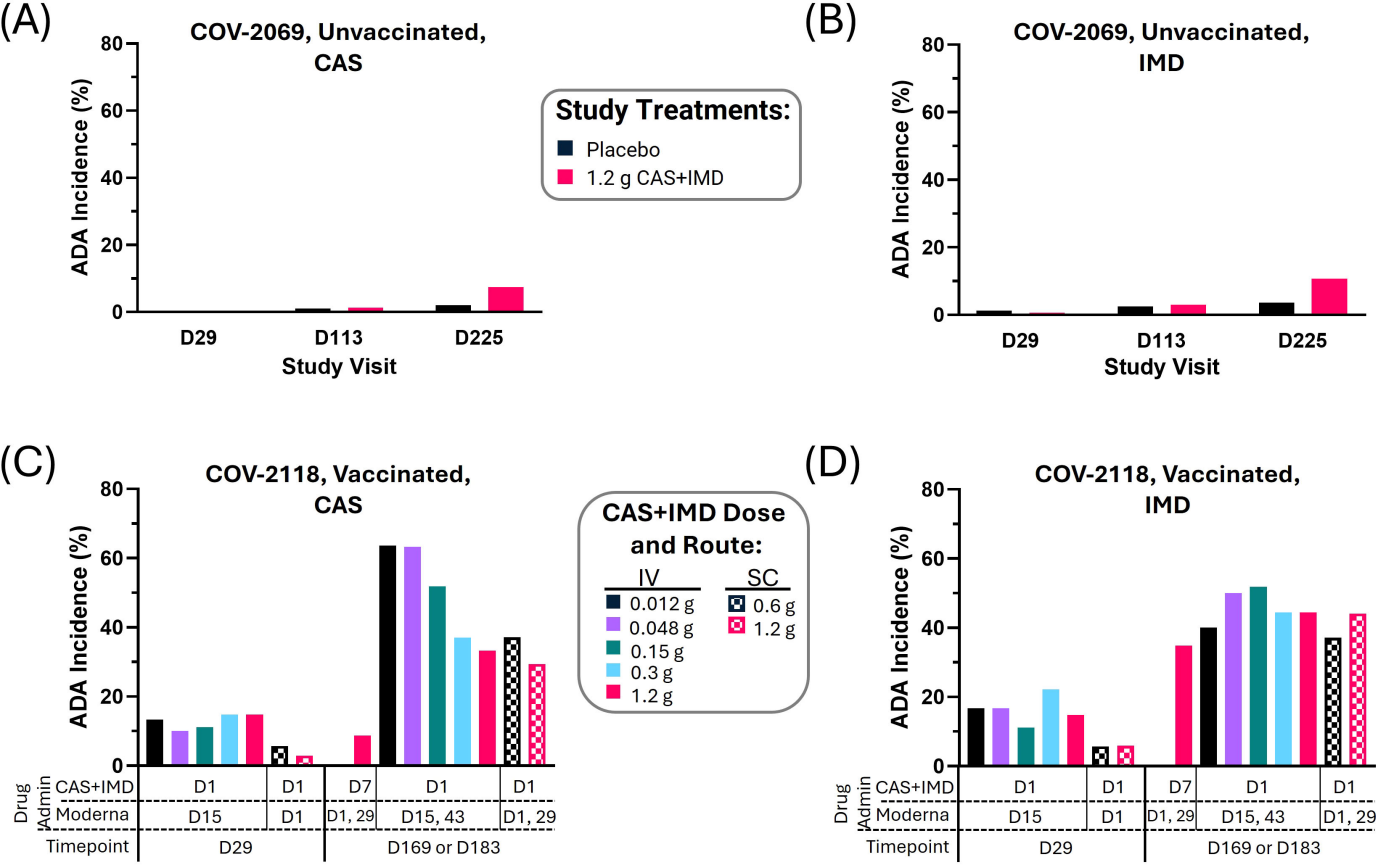
Compared to unvaccinated study participants in COV-2069, increased immunogenicity to the CAS+IMD mAbs was observed in a clinical study (COV-2118) investigating vaccine-mAb interaction when participants were vaccinated with an mRNA-based SARS-CoV-2 vaccine. Study COV-2069 anti-drug antibody (ADA) incidence to CAS **(A)** and IMD **(B)** was low at study day 29 (D29), D113, and D225 for unvaccinated participants. The COV-2069 ADA analysis included 972 and 995 participants who were administered placebo or casirivimab plus imdevimab (CAS+IMD), respectively. Higher ADA incidence to CAS **(C)** and IMD **(D)** was observed in Study COV-2118 at D29 and either D169 or D183 (later timepoint varied per study group). The COV-2118 ADA analysis included a total of 233 participants who were administered both CAS+IMD and Moderna’s COVID-19 vaccine in one of 7 different study groups where group size was 23–25 participants. In this study, the timing of CAS+IMD administration and (initial) vaccine dose varied per study group (indicated below the x-axis) as well as the dose and route of CAS+IMD administration (legend). Intravenous (IV), subcutaneous (SC)).

Compared to the relatively low cumulative immunogenicity incidence in unvaccinated participants in COV-2069 (<8% (75/995) for CAS and <11% (106/995) for IMD) (5) (**Figures 1A, B**), the ADA incidence in vaccinated participants in COV-2118 was more than 5-fold higher (up to 63% (19/30) for CAS and 52% (14/27) for IMD for some treatment groups) (**Figures 1C, D**). When analyzed cumulatively only up to and including the indicated sample timepoint, the ADA incidence through D29 and at the later timepoint was higher in vaccinated participants and increased over time (**Figure 1**). For one group, the administration of CAS+IMD occurred 6 days (i.e., D7) after the first dose of vaccine and the immunogenicity to CAS and IMD differed: the immunogenicity incidence to CAS remained low (8.7% (2/23)) (**Figure 1C**) even though anti-IMD Abs became elevated (34% (8/23) (**Figure 1D**), as observed with other groups. This effect was not observed with other groups who received the same dose of 1.2 g CAS+IMD via the same or a different route of CAS+IMD administration. The specific level of observed ADA incidence to CAS and IMD varied per dose-based group, but no correlations or trends of CAS+IMD dose and incidence were identified (**Figure 1**). Similarly, the route of CAS+IMD administration was not associated with differences in ADA incidence (**Figure 1**).

The dramatic increase in immunogenicity incidence in participants vaccinated with SARS-CoV-2 mRNA vaccine in Study 2118 was unexpected because low immunogenicity incidence to CAS+IMD was observed previously in multiple other clinical studies (5). To confirm this result, a reanalysis of prior CAS+IMD studies was performed to examine immunogenicity to CAS+IMD in participants who were vaccinated after the efficacy assessment period and while continuing to participate in the CAS+IMD studies.

### Immunogenicity incidence to mAbs varied with vaccine type

**Figure 2:**
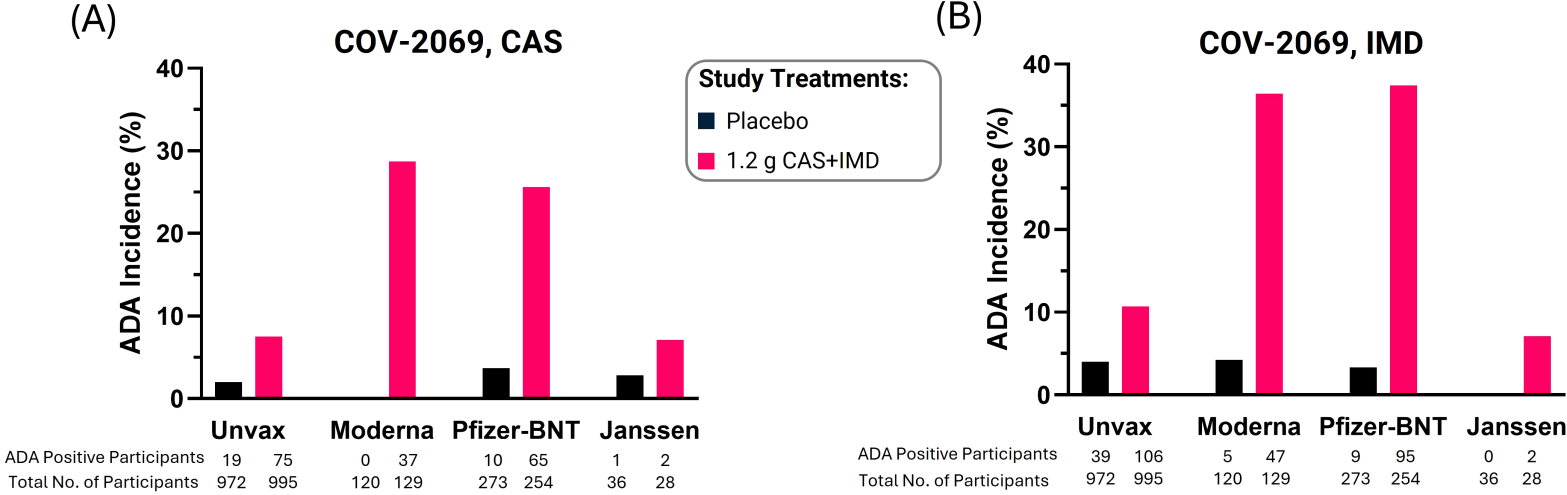
Retrospective analysis of CAS+IMD prophylaxis study (COV-2069) revealed increased ADA incidence to mAbs in SARS-CoV-2 mRNA vaccinated participants. Incidence (%) of ADA to CAS **(A)** and IMD **(B)** that developed in participants administered a single dose of placebo or 1.2 g CAS+IMD; data is grouped based on COVID-19 vaccination status and by vaccine manufacturer. Unvaccinated (unvax), Pfizer-BioNTech (Pfizer-BNT).

In Study COV-2069, participants were permitted to receive COVID-19 vaccination while still remaining part of the CAS+IMD clinical trial; specifically, the use of approved or emergency use authorized (EUA) vaccines were allowed after the efficacy assessment period (EAP) (i.e., vaccination was allowed during the study follow-up period). As a result, this study provided an opportunity to examine the impact of vaccination on immunogenicity to CAS and IMD within the study’s population through end of study, i.e., D225 (**Figure 2**). Furthermore, since multiple different COVID-19 vaccines were available while Study COV-2069 was ongoing, this study supported an investigation of the effect of different vaccines on immunogenicity to CAS+IMD (**Figure 2**). In study COV-2069, all participants received the same CAS+IMD dose or placebo. This allowed for a more robust comparison between vaccinated and unvaccinated groups because the number of participants with the same CAS+IMD dose was larger than in Study COV-2118.

As expected, immunogenicity was low (<5%) in participants who did not receive CAS+IMD (i.e., placebo study treatment), regardless of vaccination status. In participants who received either Moderna’s or Pfizer-BioNTech’s mRNA-LNP-based COVID-19 vaccines, there was a significant increase in ADA incidence to CAS and IMD (>35%) as compared to unvaccinated participants (<11%) at the late timepoint (i.e., D225). Interestingly, a parallel increase in ADA incidence to CAS and IMD was not observed in participants vaccinated with Janssen’s adenovirus-based COVID-19 vaccine. It should be noted that the Janssen vaccine lacks an adjuvant (29) and is reported to have 10-fold lower concentrations of spike in serum than with an mRNA-LPN-based COVID-19 vaccine (17). These data suggest the type of the vaccine and/or amount of vaccine-induced target protein impacted the CAS+IMD immunogenicity incidence that developed over the extended observation time.

For participants administered CAS+IMD, a minority (42%) received COVID-19 vaccination during the COV-2069 study. However, across all active-drug participants that developed an ADA response (i.e., unvaccinated participants + vaccinated participants with ADA), more than half [58% (104/179) for CAS and 58% (144/250) for IMD] of these participants were vaccinated. Therefore, while vaccinated participants comprised a smaller portion of overall participants, they constituted the major contribution to the study’s immunogenicity incidence when all participants were grouped together.

### Low NAb incidence and low titer ADA are observed independently of vaccination status

A mature and clinically impactful humoral immune response to biotherapeutics is often characterized by high titer ADA and high incidence of NAb-positivity (30, 31). As previously reported, immunogenicity to CAS+IMD was not associated with any clinical impact on PK, safety, or efficacy in unvaccinated participants (5). Nonetheless, Study 2069 contained participants with and without vaccination and therefore, ADA titer and NAb incidence for CAS+IMD could be examined in participants with and without vaccine-induced target (i.e., systemic spike protein).

The CAS NAb positivity rate was very low for all participants, with a minor difference in NAb incidence between participants with different vaccination status: 1% (9/995) NAb incidence in unvaccinated and 3% (12/409) NAb incidence in vaccinated participants (**Supplementary Table 1**). Generally, the incidence of NAb positivity to IMD was also low (12%) in the study overall, although it was higher than observed for NAb incidence to CAS (1%). More specifically, vaccinated participants had higher NAb positivity (22%, 92/411) against IMD as compared to unvaccinated participants (7%, 74/995) (**Supplementary Table 1**). Because most participants did not develop NAb responses, it appears that even when a humoral response developed against CAS and IMD, it did not fully mature to have neutralizing capabilities.

Importantly, for both CAS and IMD, the range of ADA titers observed for all participants was similar, regardless of vaccination status. Furthermore, a large majority (89-92%) of the ADA which occurred through the end of study was low titer in all participants, both vaccinated and unvaccinated (**Supplementary Figure 1**). These findings are representative of >10 clinical studies across the CAS+IMD drug development program where neither high magnitude ADA nor high incidence of NAbs was observed for CAS+IMD regardless of participant vaccination status.

### No change in ADA for unrelated mAbs after mRNA-based vaccination

One plausible explanation of the increased ADA in mRNA-LNP vaccinated participants is that the LNP and/or polyethylene glycol (PEG) components of the mRNA vaccines may act as non-specific adjuvants. However, this non-specific mechanism would not be limited to CAS+IMD drug product and therefore would be expected to impact ADA rates for other protein therapeutics administered in clinical trials (and clinical practice) conducted during and after the COVID-19 pandemic. We examined 2 late phase clinical trials for a different mAb therapeutic that did not target an infectious disease antigen. Each of these trials were conducted during the COVID-19 pandemic with more than 900 participants administered either placebo (~33%) or active drug (~66%), per study (unpublished data). More than 20% of participants received at least 1 dose of COVID-19 vaccine while on study. For active drug-treated participants, there was <2% ADA incidence observed and all of those were low titer responses. In addition, there are no publicly available data for other protein therapeutics indicating that administration of any type of vaccine (including LNP/PEG-based vaccines) is a significant risk factor for ADA development. Finally, LNP-encapsulated siRNA products have similarly low ADA rates as non-encapsulated siRNA drugs (32). Taken together, these data suggest that LNP and/or PEG are not likely acting as a non-specific adjuvant to stimulate ADA development to mAbs.

### Formation of large, stable mAb:target immune complexes

Since COVID-19 vaccinated individuals have spike trimer present in systemic circulation, an alternative hypothesis is that upon CAS+IMD administration, high molecular weight ICs of CAS+IMD and spike trimer are formed, which may lead downstream to development of anti-CAS and anti-IMD antibodies. The anti-TNF therapeutics adalimumab and infliximab have elevated ADA incidence, which has been attributed to the formation of large drug:target complexes (14–16). It is possible that a related phenomenon is occurring with CAS+IMD in mRNA-vaccinated participants where spike trimer has been expressed.

To test the hypothesis that large mAb:spike timer ICs can form, SV-AUC, which provides first principle hydrodynamic information about the size, shape, and interactions of macromolecules in solution, was adopted to characterize the complexes that form when CAS and IMD are incubated with recombinant spike trimer *in vitro*.

**Figure 3:**
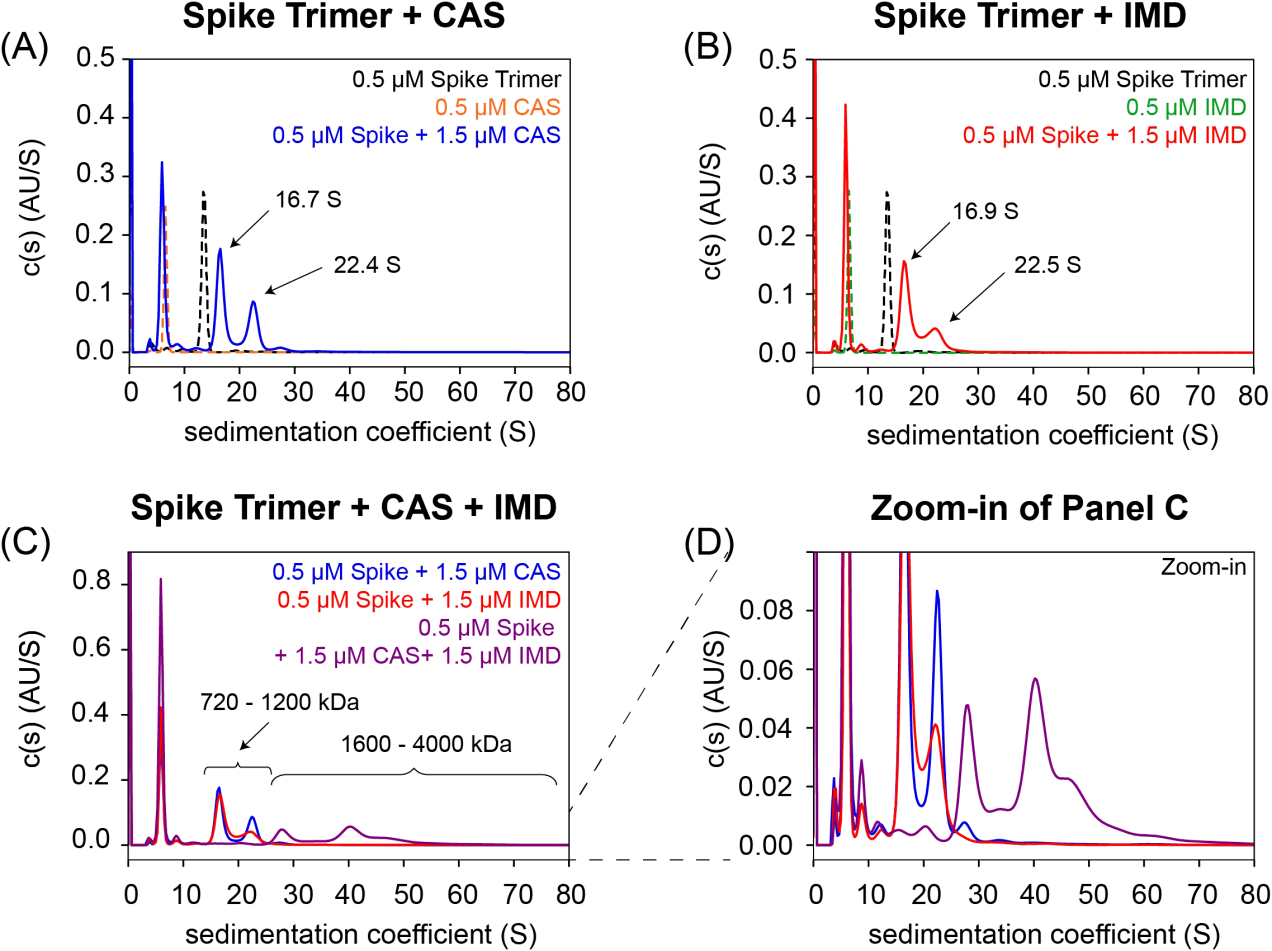
Simultaneous incubation of CAS+IMD in molar excess with target (i.e., SARS-CoV-2 spike trimer) results in formation of large, stable immune complexes *in vitro*. The distribution of sedimentation coefficients (c(s)) derived from SV-AUC experiments are depicted for recombinant spike trimer, CAS, and IMD alone and their complexes when combined. **(A)** c(s) distributions are overlaid of spike trimer (black), CAS (orange), and the 1:3 spike trimer: CAS complex (blue). **(B)** c(s) distributions are overlaid of spike trimer (black), IMD (green), and the 1:3 spike trimer: IMD complex (red). **(C)** c(s) distributions are overlaid of the 1:3 spike trimer: CAS (blue), 1:3 spike trimer: IMD (red), and the 1:3:3 spike trimer: CAS: IMD complex (purple). **(D)** Zoomed-in view of the c(s) distributions shown in panel C, emphasizing the higher order complexes (~1600 to 4000 kDa) formed by potential cross-linking of multiple spike trimers by CAS+IMD simultaneously.

As controls, each individual protein was evaluated by SV-AUC to determine the purity and oligomeric state of the major observed species. The sedimentation profile of each individual mAb (CAS and IMD) exhibited a single, predominant peak at 6.6 S, consistent with a monomeric mAb (theoretical molar mass = ~145 kDa), while the spike trimer exhibited a single predominant peak at 13.5 S, consistent with the larger expected size of a trimeric spike protein (theoretical molar mass ~400 kDa). The interactions of each individual mAb with the spike trimer were also evaluated (CAS, **Figure 3A** and IMD, **Figure 3B**). When a molar excess of each individual mAb was incubated with spike trimer (0.5 µM:1.5 µM spike trimer: mAb), a similar sedimentation profile was observed for each sample with two discrete peaks sedimenting faster (~17 S and ~22 S) than the individual protein components consistent with the formation of mAb:spike trimer complexes. Based on the sedimentation coefficients, the calculated molar mass range of the observed peaks was ~720–1200 kDa suggesting that they correspond to complexes formed between 1–2 mAb molecules binding to either one (peak at ~17S) or two (peak at ~22S) spike trimer molecules (**Figures 3A, B**). Most importantly, under these conditions neither CAS nor IMD individually formed extended, higher order complexes with spike trimer. However, when both mAbs (CAS+IMD) were incubated simultaneously in molar excess with spike trimer, very large, heterogeneous complexes were formed ranging from ~1600 to 4000 kDa in molar mass (**Figure 3C**), suggesting the presence of extended mAb:spike trimer lattices that contain both CAS and IMD. These data clearly indicate that CAS+IMD can engage multiple spike trimer molecules simultaneously, forming extended higher order complexes.

## Discussion

The CAS+IMD drug development program provided a distinct opportunity to investigate the impact of the presence or absence of target protein on immunogenicity to a mAb therapeutic. Due to the nature of the SARS-CoV-2 pandemic, clinical studies were initially conducted in participants who were unvaccinated and therefore had no exogenous target present systemically (or at very low levels in infected patients). Once the vaccines were available, CAS+IMD study participants were allowed to be vaccinated after completing the efficacy assessment period. In addition, a clinical study (i.e., COV-2118) was specifically conducted to understand the impact of these mAbs on the COVID-19 vaccine response (19). In both scenarios for vaccinated study participants, CAS+IMD was present in blood concurrently with the spike protein, which was expressed transiently (maximum expression at 7 days) after each vaccination (17, 33). The immunogenicity data were striking, with development of ADA to the mAbs at rates >3-fold higher in vaccinated participants. The most likely mechanism that could explain this result is the generation of higher molecular weight target:drug ICs that allow increased immune surveillance and uptake by macrophages and dendritic cells, resulting in elevated ADA incidence (16, 18).

### Immune complexes

Because the SARS-CoV-2 viral spike protein forms a trimer, and because CAS and IMD bind to different regions of the angiotensin-converting enzyme 2 (ACE-2) receptor binding domain (34), large CAS+IMD:spike trimer ICs could potentially form. This was demonstrated with *in vitro* experiments using recombinant spike trimer and CAS+IMD, where large complexes of both mAbs and spike trimer were identified (**Figures 3C, D**). There are multiple reported examples where aggregates or HMW impurities can increase immunogenicity (11–13, 18). Interestingly, formation of large ICs between TNFα and either adalimumab or infliximab has been invoked as a potential explanation for the increased immunogenicity of these mAbs compared to other anti-TNFα products (14, 15). Large ICs likely increase the uptake of the mAbs by antigen-presenting cells and can also directly cross-link B-cell receptors, activating B-cells independently of T-cells (16).

In addition, concentrations of spike protein in serum using an mRNA-LNP-based COVID-19 vaccine are reported to be 10-fold greater than those observed with an adenovirus-based vaccine (17). The presence of significantly more target protein (i.e., spike protein), and consequently the greater potential to form mAb:target protein ICs, is consistent with the greater ADA incidence observed in participants vaccinated with mRNA-based products (i.e., Moderna and Pfizer-BioNTech) compared to participants vaccinated with an adenovirus-based product (i.e., Janssen) (**Figure 2**).

As a post-marketing commitment for a different anti-infective mAb, raxibacumab, an anti-anthrax therapy, a combination study of mAb and vaccination was conducted in healthy subjects (35). In the trial, subjects were administered Anthrax Vaccine Adsorbed (AVA) alone or a combination of mAb (i.e., raxibacumab) immediately followed by AVA. Similar to the reported results for CAS+IMD on COVID-19 vaccination, raxibacumab did not affect the immunogenicity of the vaccine but did elicit increased immunogenicity incidence against the mAb therapeutic (i.e., from 0% in unvaccinated to 12.9% in AVA-vaccinated) (35). Again, the presence of target protein upon vaccination may have resulted in increased IC formation and greater immune surveillance of the mAb. However, mRNA-LNP vaccines induce a large amount of systemic target which may account for the substantially higher anti-mAb response in the vaccinated participants that were also administered with CAS+IMD (17).

### ADA responses to CAS+IMD were low magnitude

Anti-CAS and anti-IMD ADA titers remained low in vaccinated participants (**Figure 3**), and there was no observed impact on PK and therefore these low magnitude responses would likely not impact efficacy of CAS+IMD. Furthermore, neutralizing responses to CAS+IMD were generally not observed, suggesting the immune response did not continue to mature. This may have been because components of the ICs were limiting: only a single dose of CAS+IMD was administered and only 2 doses of the COVID-19 vaccination occurred during the maximum 225 days of study participant observation. It is possible that a repeat administration of CAS+IMD in vaccinated participants could have resulted in development of higher magnitude ADA. However, repeat administration in the absence of vaccination did not cause elevated ADA incidence as lower drug exposure was shown to be the primary driver of ADA development (5). Importantly, in contrast to the ADA response to CAS+IMD, the immune responses to the spike protein in vaccinated participants remained robust, high titer, and neutralizing (28). This indicated that these participants were capable of generating a vigorous humoral response to a foreign antigen, but that the fully human mAbs CAS+IMD likely maintained some level of immune tolerance.

## Conclusion

This is the first reported observation of high incidence of ADA to mAb therapeutics (CAS+IMD) when individuals were also administered a 2-dose regimen vaccine (COVID-19 vaccine) against the same exogenous target antigen (i.e., SARS-CoV-2 spike protein). The strongest association between COVID-19 vaccine and CAS+IMD was in participants who received an mRNA-LNP-based vaccine, likely due to formation of large, stable ICs consisting of mAb(s) and vaccine-induced spike which led to increased immune system surveillance and thus increased ADA. Despite the increased ADA incidence, the impact of the ADA was not observed directly or indirectly on PK, safety, or efficacy. Collectively, these data help elucidate the mechanism of ADA development and indicate a need for monitoring future interaction studies in participants who are administered both vaccines and antibody therapeutics.

## Data Availability

Qualified researchers may request access to study documents (including the clinical study report, study protocol with any amendments, blank case report form, statistical analysis plan) that support the methods and findings reported in this manuscript. Individual anonymized participant data will be considered for sharing 1) once the product and indication has been approved by major health authorities (e.g., FDA, EMA, PMDA, etc) or development of the product has been discontinued globally for all indications on or after April 2020 and there are no plans for future development 2) if there is legal authority to share the data and 3) there is not a reasonable likelihood of participant re-identification. Submit requests to https://vivli.org/.
