# Supplementary material for: The role of large immune complexes in anti-drug antibody development: a case study of anti-SARS-CoV-2 antibody therapeutics and co-administered mRNA vaccine
Source: Front Immunol. 2026 Mar 10;17:1769163. doi: 10.3389/fimmu.2026.1769163 (PMC13008684; doi:10.3389/fimmu.2026.1769163)
Supplement: Supplementary file 1 [file Supplementaryfile1.docx]

**Supplemental Figure 1.**


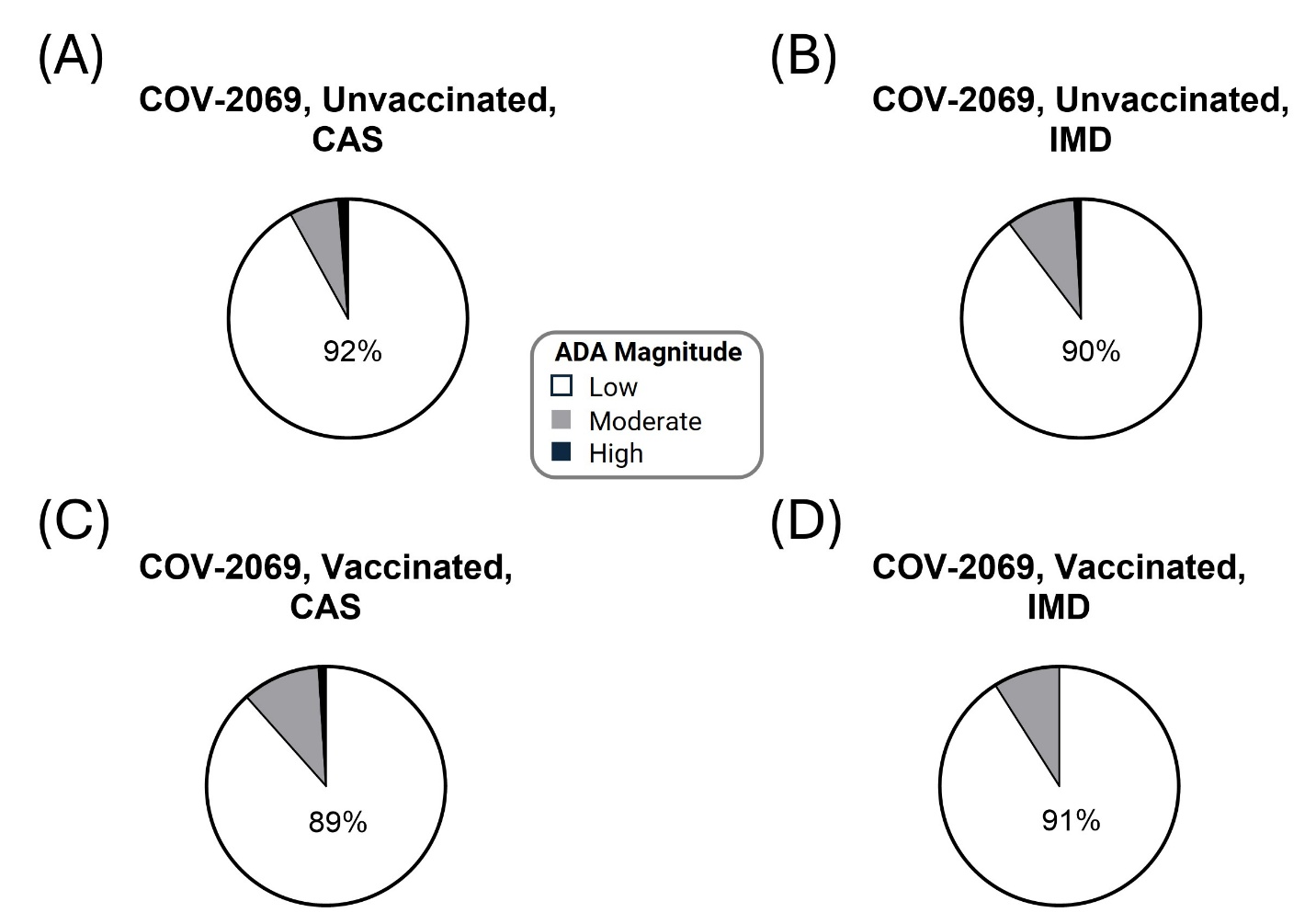


Supplemental Figure 1. Low titer ADA was observed in Study COV-2069 participants who were administered CAS+IMD (1.2 g) irrespective of SARS-CoV-2 vaccination status (unvaccinated, n = 995; vaccinated, n = 409). Reported maximum ADA titer per participant through study day 225 for casirivimab (CAS) (A and C) and imdevimab (IMD) (B and D) in unvaccinated (A and B) or vaccinated (C and D) participants. The magnitude of the observed ADA responses were categorized per participant as low (if titer < 1,000, white), moderate (if 1,000 ≤ titer ≤ 10,000, grey), or high (if titer > 10,000, black).
