# Supplementary material for: The role of large immune complexes in anti-drug antibody development: a case study of anti-SARS-CoV-2 antibody therapeutics and co-administered mRNA vaccine
Source: Front Immunol. 2026 Mar 10;17:1769163. doi: 10.3389/fimmu.2026.1769163 (PMC13008684; doi:10.3389/fimmu.2026.1769163)
Supplement: Supplementary file 2 [file Table1.docx]

**Supplemental Table 1.**

|  | COV-2069, CAS | |  | COV-2069, IMD | |
| --- | --- | --- | --- | --- | --- |
|  | Unvaccinated | Vaccinated |  | Unvaccinated | Vaccinated |
| NAb negative | 99% (986/995) | 97% (397/409) |  | 93% (921/995) | 78% (317/409) |
| NAb positive | 1% (9/995) | 3% (12/409) |  | 7% (74/995) | 22% (92/409) |

Supplemental Table 1. Incidence [%, (n/N)] of neutralizing antibodies (NAb) to CAS and IMD remains low over time. Study COV-2069, Cohort A, participants who were uninfected at enrollment/baseline. Participants positive for NAb (n), participants positive or negative for NAb (N).
